# III-nitride core–shell nanorod array on quartz substrates

**DOI:** 10.1038/srep45345

**Published:** 2017-03-27

**Authors:** Si-Young Bae, Jung-Wook Min, Hyeong-Yong Hwang, Kaddour Lekhal, Ho-Jun Lee, Young-Dahl Jho, Dong-Seon Lee, Yong-Tak Lee, Nobuyuki Ikarashi, Yoshio Honda, Hiroshi Amano

**Affiliations:** 1 Institute of Materials and Systems for Sustainability (IMaSS), Nagoya University, Nagoya, 464-8603, Japan; 2 School of Electrical Engineering and Computer Science, Gwangju Institute of Science and Technology (GIST), Gwangju, 61005, Republic of Korea; 3 Department of Electrical Engineering and Computer Science, Nagoya University, Nagoya, 464-8603, Japan; 4 Akasaki Research Center (ARC), Nagoya University, Nagoya, 464-8603, Japan

## Abstract

We report the fabrication of near-vertically elongated GaN nanorods on quartz substrates. To control the preferred orientation and length of individual GaN nanorods, we combined molecular beam epitaxy (MBE) with pulsed-mode metal–organic chemical vapor deposition (MOCVD). The MBE-grown buffer layer was composed of GaN nanograins exhibiting an ordered surface and preferred orientation along the surface normal direction. Position-controlled growth of the GaN nanorods was achieved by selective-area growth using MOCVD. Simultaneously, the GaN nanorods were elongated by the pulsed-mode growth. The microstructural and optical properties of both GaN nanorods and InGaN/GaN core–shell nanorods were then investigated. The nanorods were highly crystalline and the core–shell structures exhibited optical emission properties, indicating the feasibility of fabricating III-nitride nano-optoelectronic devices on amorphous substrates.

Over the last several decades, research in III-nitride epitaxy has focused on achieving large-scale two-dimensional (2D) layers with high crystallinity for efficient light-emitters that operate over a wide wavelength range (ultraviolet to infrared)^1^,^2^. InGaN/GaN-based light-emitting diodes (LEDs) are usually grown on sapphire substrates, which have similar lattice properties to GaN and are thermally stable under induced strain. Other candidate substrates for III-nitride epitaxy have received less attention^3^. Although a few other substrates such as Si and SiC have been applied in particular electronic devices and vertical architectural devices, high-quality GaN on amorphous substrates is desired for future applications requiring transparency and flexibility^4^,^5^. Recently, the crystal quality and preferential orientation of GaN on amorphous layers have been sufficiently improved for feasible optoelectronic device operation^6^,^7^,^8^. All of these breakthroughs are based on a common concept; restricting the degrees of freedom of grown GaN using a pre-orienting layer (POL) or external mask frames. In particular, local epitaxy (on scales of a few micrometres or less) has achieved fully flexible LEDs by a transfer technique^9^,^10^.

Undoubtedly, for successful epitaxy on amorphous substrates, we must appropriately select the alternative substrates and POL. To form reproducible single crystals in the growth system, these substances must fulfil several important criteria: (1) thermal stability without deformation and contamination at high growth temperature; (2) polished flat surface for subsequent material growth; (3) little in-plane lattice mismatch of unit cells for stacking the upper GaN layer; and (4) no severe chemical reaction between the POL and the substrate, which would lead to voids, cracks and delamination. Hence, the candidate elements of alternative amorphous substrates and POLs are limited (see Supplementary Table S1). In this study, we mainly consider epitaxy on a quartz (fused silica) substrate without a metal-based POL. To achieve high-quality GaN and device fabrication on amorphous substrates, we must also consider various physical factors such as the glass-transition temperature, thermal expansion coefficient, thermal conductivity, crystal quality, optical property and electrical contact method (see Supplementary Table S2). However, the inability to solve all of the arising problems has limited the performance of 2D-layer-based devices. Three-dimensional (3D) building blocks using nanoscale local epitaxy promise to overcome the above obstacles. Indeed, GaN nanowires grown on amorphous layers by molecular beam epitaxy (MBE) have shown high crystal quality and superior optical properties^11^,^12^. Moreover, as their diameter reduces, 3D nanowires are also less affected by the additional strain induced by the thermal expansion coefficient mismatch than 2D layers and therefore remain crack-free during the cooling process^13^. In addition, some large-area amorphous substrates with high-temperature (>1000 °C) endurance, such as quartz and alumina, are easily available at low cost. These substances can be combined with III-nitride nanostructures that have several desirable properties: filtered dislocations, suppression of the quantum-confined Stark effect, enhanced indium incorporation, tuneable emission spectrum by adjusting the diameter and/or external bias and wide emission area^14^,^15^,^16^,^17^,^18^. Recent progress of the transfer method facilitates for 3D building blocks to form cathodes and anodes for electrical devices and to be bonded to a metallic substrate for heat dissipation^19^,^20^.

The most difficult task in local epitaxy on amorphous layers is retaining a geometrically ordered architecture, as the grown structures lack an ordered substrate lattice^21^. The uniformity of the material and optical properties of the subsequent device fabrication also require an ordered formation^21^ To achieve ordered GaN structures on amorphous substrates, researchers have deposited covering layers with hexagonal lattice structures, such as titanium and graphene, which induce preferential orientation of the upper epilayers^6^,^8^,^22^. Other POL candidates are listed in Supplementary Table S1. Position-controlled 3D structures on POLs have been grown by selective-area growth (SAG)–adatom adsorption, which favours the nuclear sites of the opening area on the mask^6^,^23^. The recent development of nanoimprints has eased the task of nanoscale patterning on flat surfaces, even over large areas^24^. In this technique, precise patterning from the mould of a nanoimprint mask requires an appropriately flat surface. To date, GaN layers grown on amorphous substrates by metalorganic chemical vapor deposition (MOCVD) generously have rough surfaces^25^. On the other hand, MBE can obtain relatively smooth surfaces by precisely controlling the atomic layers, forming a homogeneous layer over the whole surface^26^.

To solve the above problems, we here combine MBE and MOCVD with nanoscale local epitaxy (i.e. nanoscale SAG) to improve the crystal quality of GaN on amorphous layers. Recently, we reported that GaN layers can evolve on amorphous substrates through microscale SAG with striped openings, where a relatively smooth GaN buffer layer was grown by MBE without a POL^27^. However, although the crystal quality in the previous stage is improved, it is still far from realising optoelectronic devices on amorphous substrates due to the high level of structural defects. It is necessary to reduce the defects to able to grow device structures on amorphous substrates including active layers. Thus, in this study, we control the morphology and grains on nanoscale hole templates. The coalescence of adjacent GaN structures is suppressed by anisotropic growth of pulsed-mode MOCVD^28^, thereby leading an elongation of GaN nanorods (NRs). This growth is effective even on low-quality and thin templates^29^, suggesting its applicability to epitaxy on amorphous substrates. Owing to the relevant uniformity of GaN NR array, InGaN/GaN core–shell layers are grown and their structural and optical characteristics are analysed to facilitate potential device applications.

## Results and Discussion

**Figure 1:**
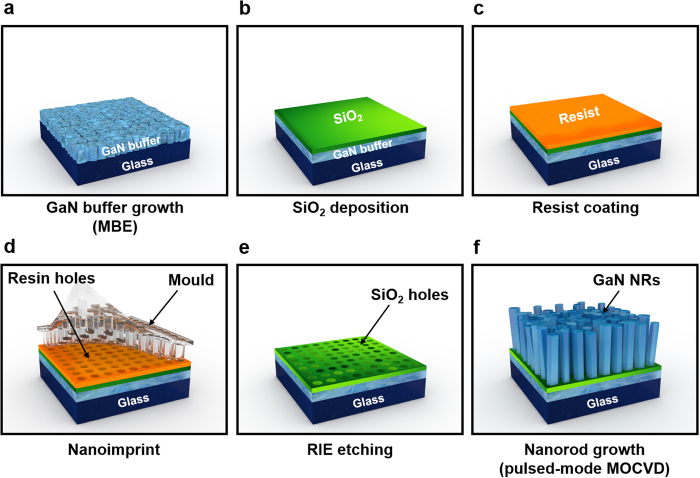
Schematic of the growth procedure for GaN NRs on quartz substrates. (**a**) GaN buffer growth by MBE, (**b**) SiO_2_ deposition, (**c**) resist coating, (**d**) thermal nanoimprint, (**e**) RIE dry etching and cleaning and (**f**) NR growth by pulsed-mode MOCVD.

**Figure 2:**
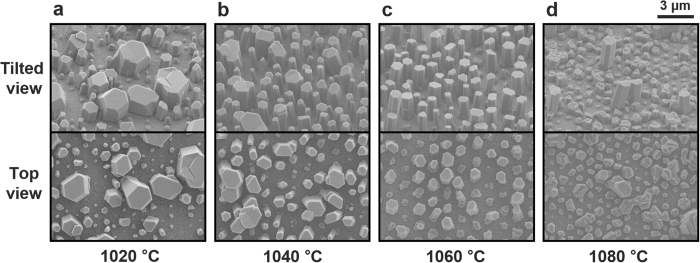
SEM images of GaN nanostructures grown at different temperatures. (**a**) 1020 °C, (**b**) 1040 °C, (**c**) 1060 °C and (**d**) 1080 °C. Upper and lower images are tilted views (45°) and top views, respectively.

**Figure 3:**
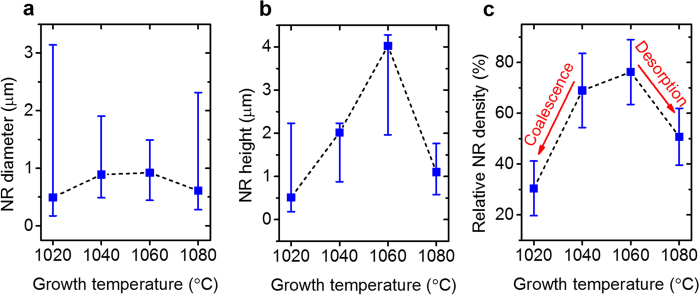
Statistical distribution of GaN NRs as functions of growth temperature. (**a**) Diameter (**b**) height and (**c**) relative density.

An overview of the experimental procedure is given in Fig. 1. To investigate the morphology change of GaN NRs, we investigated several growth temperatures in the range 1020–1080 °C. The upper and lower panels of Fig. 2 show tilted and top-view scanning electron microscopy (SEM) images, respectively, of the grown GaN nanostructures. At 1020 °C, most of the micro scale grains were coalesced because pulse-mode growth favors the lateral direction at low growth temperature^30^. When the growth temperature was increased to 1060 °C, the coalesced micro-scale grains became smaller and the density of the GaN NRs increased (Fig. 2b and c). At 1080 °C, most of the GaN NRs failed to elongate (Fig. 2d) and their top surfaces were randomly grooved with protruded structures. This indicates that strong Ga desorption at high temperature suppressed the adsorption of Ga adatoms on the *c*-plane top surface^31^. The statistical diameter and height distributions of the grown GaN NRs are presented in Fig. 3a and b, respectively. The error bars extend from the minimum to the maximum lengths. The average values were obtained by a Gaussian fitting of the acquired histogram data. The opening hole diameter was 190 nm for the GaN NRs grown at 1020 and 1080 °C and 460 nm for those grown at 1040 and 1060 °C. Although the former average diameter was approximately half the latter diameter, the error bars reduced as the growth temperature increased from 1020 to 1060 °C and increased at the highest growth temperature (1080 °C). The increased diameter distributions clearly related to the coalescence behaviours observed in the SEM images. The average heights of the NRs behaved oppositely to the average diameters over the same temperature range (Fig. 3b); that is, the GaN NRs grown at 1060 °C showed the highest average height of 4.03 μm. The axial growth rate increased up to ~73 nm/min with increasing growth temperature. The relative density of the NRs is plotted in Fig. 3c. Because the SAG was performed on a patterned hole array, we defined the relative NR density as the number of grown NRs divided by the number of maximum opening holes over the same area. The average relative NR density peaked at 76.2% at 1060 °C. At lower temperatures, the relative NR density was reduced by coalescence, which decreased the number of grown NRs. The density reduction at higher temperatures is attributed to insufficient filling of the opening holes under the strong Ga desorption effect. Indeed, the growth temperature critically affected the morphology and density of the NRs. The optimised growth temperature was ~50 °C higher on the quartz substrates than in our previous pulsed-mode MOCVD on common sapphire substrates^30^. This result might reflect the lower heat transfer of the quartz substrates due to their lower thermal conductivity (~1.4 Wm^−1^ K^−1^) than sapphire and their large thickness (~1 mm).

### Crystallographic alignment of GaN NRs

**Figure 4:**
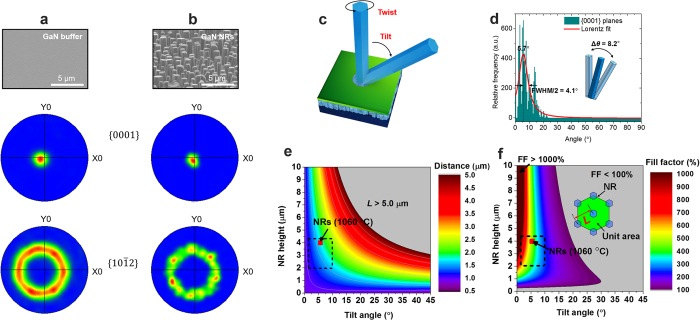
EBSD results of grown GaN structures. (**a**) GaN buffer and (**b**) GaN NRs, showing the measured SEM images (top) and the {0001} and 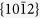 hexagonal pole figures (centre and bottom, respectively). X0 and Y0 denote the rolling and transverse directions, respectively. (**c**) Schematic of inclined NRs with a tilt and a twist. (**d**) Relative frequency of signals versus angle in {0001} hexagonal pole figures. Contour maps of height versus tilt angle of the NRs, showing (**e**) the acceptable pitch-to-pitch distances (*L*) of the hole openings (coloured regions) and (**f**) fill factor. The red squares indicate the average values of the height and tilt angle of GaN NRs grown at 1060 °C and the black dashed rectangles show the distributions of the height and tilt angle.

To correlate the distribution of crystal orientation between the templates and the above grown structures, we measured the electron backscatter diffraction (EBSD), as shown in Fig. 4a,b. The uppermost parts of panels a and b are representative SEM images of the GaN buffer and GaN NRs above the buffer, respectively. The relatively smooth morphology enabled the SiO_2_ hole openings during the nanoimprinting process. Subsequently, near-vertical NRs were grown on the periodically formed hole openings. The middle and bottom sections of panels a and b are hexagonal pole figures of the {0001} and {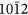 } planes, respectively. The EBSD measurement area was set to (5 × 5) μm^2^. In the {0001} planes, the GaN buffer was preferentially oriented along the out-of-plane (surface normal) direction. Conversely, the {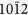} planes yielded a circular ring pattern, indicating no preferential orientation along the in-plane (surface-parallel) direction. Hence, the GaN buffer exhibited highly polycrystalline features. Similar polycrystalline features were found on the ensemble of GaN NRs, in both {0001} and {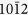} planes. The hexagonal pole figure of GaN NRs presented not only an intense single spot at around the centre of {0001} view but also a dispersed circular ring in {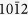} planes. Hence, the preferred orientation of GaN NRs almost identically followed that of the GaN buffer during growth evolution. However, different from the GaN buffer, the ensemble showed localised spots of {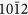} planes (see bottom part of Fig. 4b). The localised spots were mainly caused by the low sampling numbers of the enlarged grains and the shadowing effect of the 3D architectures. We emphasise that each GaN NR retained a six-fold hexagonal structure despite its enlarged size. Indeed, the combined epitaxy and nanoscale local epitaxy achieved a nearly ordered array of GaN NRs: the NRs were preferentially orientated along the surface normal direction by plasma assisted (PA)-MBE and were subsequently elongated by the pulsed-mode MOCVD with nanoscale SAG.

Although the GaN NRs were moderately ordered, their distortion angle must be quantified to avoid unwanted coalescence during the growth. As shown in Fig. 4c, the angle of an inclined NR can vary by twisting (in-plane) and tilting (out-of-plane). The deviation of the twist angle was difficult to evaluate because the in-plane surface was not structurally coherent. In addition, we aspired to grow GaN NRs with preferred orientation along the surface normal direction. Thus, we instead focused on the deviation of the tilt angle. Figure 4d shows the relative frequency of the EBSD signals in the {0001} planes as a function of angle. Lorentz fitting revealed a peak of 5.7° and a deviation of 4.1°, where the deviation is defined as half the full width at half maximum (FWHM). We can now estimate the appropriate distance of the hole mask for which all GaN NRs will be separately grown. Assuming two adjacent NRs of equivalent height, the acceptable separation of the hole mask can be simply given from


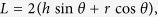


where *L, h, θ* and *r* denote the pitch-to-pitch distance of the hole mask, the NR height, the tilt angle of the NRs and the NR radius, respectively (see Supplementary Fig. S1). As an example, the average radius of GaN NRs grown at 1060 °C (461 nm; Fig. 3a) was utilised for calculation. Figure 4e shows a contour map of the pitch-to-pitch distance of the hole mask with fixed r (461 nm), indicating the dependence on tilt angle and height. To clearly visualise the region of interest (dashed rectangle in Fig. 4e), the area corresponding to L above 5.0 μm was arbitrarily excluded (grey area in Fig. 4e). Note that because the estimated distance range (>1.50 μm) slightly exceeds the utilised distance (1.38 μm), adjacent NRs could coalesce in the present study. Nevertheless, the broad statistical height and tilt angle distributions of the NRs (dashed rectangle in Fig. 4e) ensured a relatively high average density (~75%). Based on the distribution of L above with a fixed radius of 461 nm, the areal gain of 3D NRs compared with the 2D layers is next estimated for the potential realisation of inclined NR-based devices. Thus, we define a fill factor (FF), the total *m*-plane area of NRs/unit area (excluding the *c*-plane area of NRs), leading to a simple relation, 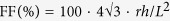. Figure 4f presents a contour plot of FF as a function of tilt angle and height. The inset of Fig. 4f is a schematic of unit area to calculate FF. The area for FF less than 100% is filled with grey colour to denote that it is less active than 2D layers. The average value and distribution of tilt angle (in Fig. 4e) and height (in Fig. 4f) of the GaN NRs grown at 1060 °C are marked by a red square and dashed rectangle, respectively. Hence, the FF of the NRs grown at 1060 °C can ideally take values >450% provided that L is carefully adjusted, as shown in Fig. 4e. Note that based on the above point in red square, FF did not increase linearly with increasing height since the value of L required to avoid coalescence increased more quickly. On the other hand, FF increased superlinearly with decreasing height based on the region below the red square in Fig. 4f. Thus, the density and active area of NRs could be controlled by adjusting L in the mask design or h via the growth time.

**Figure 5:**
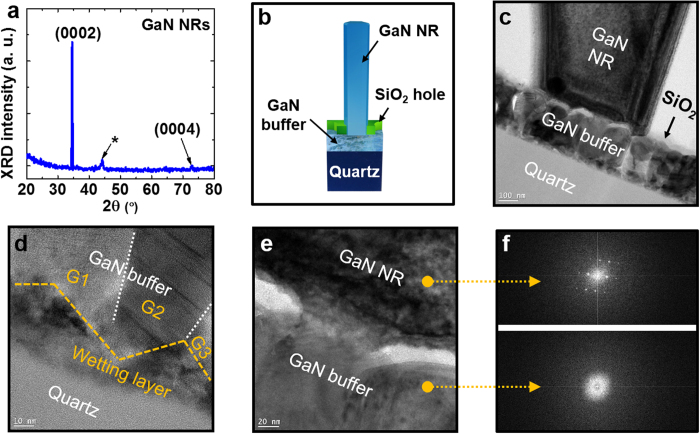
(**a**) XRD 2*θ* scans of the GaN NRs. (**b**) Schematic of a thinned specimen fabricated from an as-grown GaN NR. (**c**) Cross-sectional TEM image of the bottom region of a GaN NR. Enlarged TEM images of (**d**) GaN buffer/quartz and (**e**) GaN NR/GaN buffer interfaces. (**f**) FFT patterns of GaN NR (upper) and GaN buffer (lower).

Let us now consider the structural properties of grown GaN NRs. Figure 5a displays the X-ray diffraction (XRD) 2*θ*-scan of the GaN NRs. The dominant peak at 34.5° is attributed to (0002) orientation. The weak peak at 72.9° is attributed to (0004) orientation. Another satellite peak (asterisk) found at 44.2° is attributed to reflection from the aluminium sample holder in the XRD system. Other crystal phases such as zinc blend structures, which often occur on GaN/amorphous substrates^25^,^32^,^33^, were dramatically suppressed, indicating an ensemble of wurtzite GaN NRs. To further study the growth evolution of GaN NRs, we cut the specimen along the direction perpendicular to the substrate and performed transmission electron microscopy (TEM). Figure 5b shows the schematic of a specimen prepared from an as-grown GaN NR. The dark-field TEM image of the bottom region is shown in Fig. 5c. The diameter variation from the bottom to the top regions is attributed to the tilt angle of the GaN NR (indicating a non-perfect vertical alignment). The GaN buffer was composed of densely spaced tens-of-nanometre-scale grains, whereas the above-grown GaN NRs were enlarged and almost entirely separated. Note that no remarkable threading dislocations (TDs) penetrated the interior of the GaN NRs from the GaN buffer, indicating suppression of abrupt strain relaxations at the interface. This is an important advantage of nanoscale SAG procedures, as previous epitaxy on amorphous substrate frequently generated high density of stacking faults (SFs) and grain boundaries (GBs) in the upper-grown GaN structures^25^,^27^. Figure 5d is a more detailed TEM image of the interface between the GaN buffer and quartz substrates. Faceted wetting layers (~10–40 nm thick) appear at the interface. The grains marked G1, G2 and G3 in Fig. 5d tended to grow along the surfaces of the wetting layers. Previously, we had related the initial nucleation mechanism on the amorphous layer to Ga_2_O_3_ nanoclusters^27^. Similar growth behaviours might be induced at the interface of the GaN buffer and quartz substrates, as we used the same epitaxy system and substrates. Once the nuclei are generated on the substrates, they are governed by the Volmer–Weber mode during MBE growth, generating a nearly complete orientation of nanoscale grains along the surface normal direction. Figure 5e shows another TEM image of the interface between the GaN buffer and GaN NRs. To investigate the crystallinity, we measured the fast Fourier transform (FFT) patterns of the GaN buffer and NRs (see Fig. 5f). The bottom of the GaN NRs exhibited a single wurtzite crystal property (upper part of Fig. 5e), whereas the top of the GaN buffer (lower part) exhibited polycrystalline features with circular patterns. This single crystallisation of GaN NRs well coincides with the microstructural evolution in the structure zone model (SZM), which classically represents microstructure trends versus deposition parameters^34^. According to the SZM, the buffer GaN grown in our PA-MBE system is classified into the transition zone (*Z*_T_) using the homogeneous temperature *T*_s_/*T*_m_ of ~0.3, where *T*_s_ and *T*_m_ are the growth temperature and melting point of the materials, respectively. Hence, the primary feature of *Z*_T_ suggests that competitive grain growth led to the formation of nanoscale columnar structures with partially preferred orientation, as shown in Fig. 5c. However, we have observed that the crystallinity of the buffer GaN becomes worse as the growth temperature is abruptly increased in the MOCVD system due to thermal re-crystallisation^27^. On the other hand, GaN NRs stood out as zone II grain features in the SZM with strong selective orientation since both the surface and bulk diffusion are significantly active at such a high homogenous temperature of ~0.5^35^. Hence, the nanoscale selective epitaxy was advantageous for reducing grain boundaries compared with planar GaN layers on amorphous substrates owing to the intentionally controlled openings.

### Microstructures and cathodoluminescence of InGaN/GaN core–shell NRs

**Figure 6:**
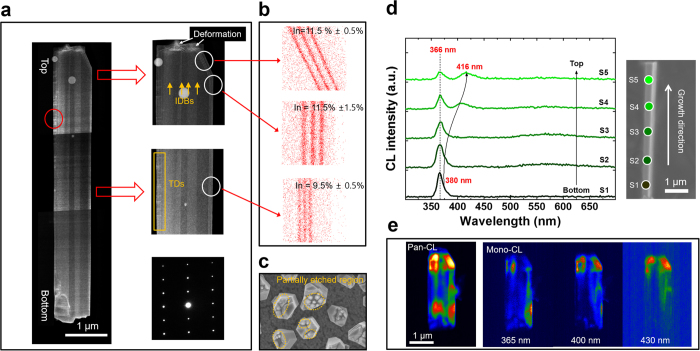
(**a**) TEM images of an out-of-plane InGaN/GaN core–shell NR. The upper and middle images on the right-hand side are enlarged images of the topmost and sidewall regions, respectively. The FFT pattern of the core GaN region is also shown (bottom right). (**b**) EDS mapping images of the semi-polar facet (upper), topmost sidewall (middle) and central sidewall (lower). (**d**) Spot-mode CL spectra collected at points S1–S5 of the SEM image. (**e**) Panchromatic (left) and monochromatic CL emission images (right) at wavelengths of 365, 400 and 430 nm.

To observe the in-plane and out-of-plane TEM views of the NRs, we prepared other specimens by separating the NRs on planar substrates (see Supplementary Fig. S2). Figure 6a shows the TEM images of an out-of-plane InGaN/GaN core–shell NR. Unlike the NR structure presented in Fig. 5c, the diameter was nearly constant over the height of ~6 μm because the cutting was precisely aligned during the specimen fabrication. Moreover, the InGaN/GaN core–shell layers conformably covered the sidewall surfaces. The only exceptional undulation appeared on the top surface of the NRs (top right of Fig. 6a). Remarkably, the brightness varied along the out-of-plane direction of the core GaN NRs. For the following reasons, we strongly believe that the section of different brightness originated from *polarity inversion* between the Ga and N polarities: (1) as confirmed by repeated FFT trials of several spots inside the GaN NRs, the (0001) or 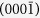 oriented wurtzite GaN structures were highly homogenous (lower right of Fig. 6a); (2) the terminated surfaces on the topmost region had flat (left side) and tilted (right side) facets, reflecting the flat top terminations of N-polar GaN and the 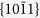 semipolar surfaces of Ga-polar GaN under the common SAG, respectively^36^,^37^,^38^; (3) when the top surfaces of grown GaN NRs were partially etched by a KOH solution (4 M at 44 °C), a mixed polarity was observed (Fig. 6c; see Supplementary Fig. S3)^38^,^39^; and (4) the observed widths of brightness contrast in Fig. 6a were almost identical to the grain size (~a few hundreds of nanometre) of the GaN buffer. We have already reported mixed polarity in the GaN buffer grown by MBE^27^. In addition, undulated surfaces are prone to polarity inversion by atomic rotation during growth of the first few GaN monolayers^40^. Hence, the inversion domain boundary (IDB) generated on the undulated mixed-polar structures critically affected the uniformity of the multiple quantum well (MQW) layers on the top surfaces.

As shown in Fig. 6a (top left), the IDBs severely deformed the MQW structures on the top surface of the InGaN/GaN core–shell layers. The growth of the polar planes largely depends on the kinetic factors during growth, which alter the surface energy^41^. In addition, these Ga- and N-polar planes accompany opposite families of semipolar planes, such as 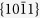 and 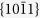 planes^42^. The slower growth of the 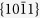 surfaces than that of the 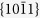 surfaces, which results from strong hydrogen passivation on N-terminated surfaces, also affects the undulated morphology of the top surface^36^. Regarding the sidewall defects, high TD densities appeared on the left side of the core–shell NRs. As these TDs were normally nucleated from the GaN core surface, they were probably affected by the relaxed strain of MQWs. Hence, the high probability of TDs on the left side than on the right side is attributed to the thicker MQWs on the left side. Similar formation of dislocations in core–shell NRs has been reported elsewhere^43^,^44^.

Another addressable issue is the varying thickness and composition of the InGaN/GaN shell structures. Based on the TEM image shown in Fig. 6a, the thickness of the wells (2.2–7.8 nm) and barriers (2.9–10.1 nm) gradually increased along the growth direction (i.e. from the bottom to top region). For the nonpolar planes, the increase of the quantum well (QW) thickness contributed to the red-shift of the luminescence peak, which empirically shifts up to ~25 nm for a QW thickness of 2–10 nm under a fixed indium composition^45^. The energy-dispersive x-ray spectroscopy (EDS) mapping images at selected positions of core–shell NRs are presented in Fig. 6b. The distributions of indium composition in the MQWs are marked for semipolar (11.5 ± 0.5%), upper (11.5 ± 1.5%) and middle regions (9.5% ± 0.5%) of core–shell NRs. The upper MQWs on the sidewalls exhibited noticeably thicker layers and higher indium composition than the lower MQW regions and had thicker layers than the semipolar regions. The latter result indicates a slower growth rate of the semipolar regions than that of the uppermost sidewalls. Figure 6d shows the spot-mode cathodoluminescence (CL) spectra of separated NRs on the Si substrates. From the bottom to the top regions (S1–S5), the emission peaks detected at the spot points gradually increased to longer wavelengths. To optically estimate the indium composition *x* of the QWs from the emission energy *E*_g_, we used the relation *E*_g_(In_*x*_Ga_1–*x*_N) = 0.64*x *+* *3.42(1 − *x*) − 2.8*x*(1 − *x*)^46^. This led to the compositional indium variation of 2.8–10.1% for the spectral range of 380–430 nm, coinciding with the trends in the EDS analysis. Thus, the energy gradient of InGaN/GaN shell layers along the growth axis is attributed to the gradual increase of both indium composition and QW thickness. Similar behaviour of energy gradient in 3D core–shell NRs has been reported elsewhere^16^,^43^,^47^. Figure 6e shows panchromatic and monochromatic CL images of similar InGaN/GaN core–shell NRs. The monochromatic CL images were obtained at emission wavelengths of 365, 400 and 430 nm. Although indium was more strongly incorporated on the topmost region of the core–shell NRs than in the lower sidewall regions, the near-bandgap emission (NBE) (365 nm) was observed throughout the core–shell NRs. Because the limited mean free path of In and Ga atoms can be extended, we speculate that the indium distribution in the InGaN QW shells could be rendered more uniform by adjusting the growth pressure.

**Figure 7:**
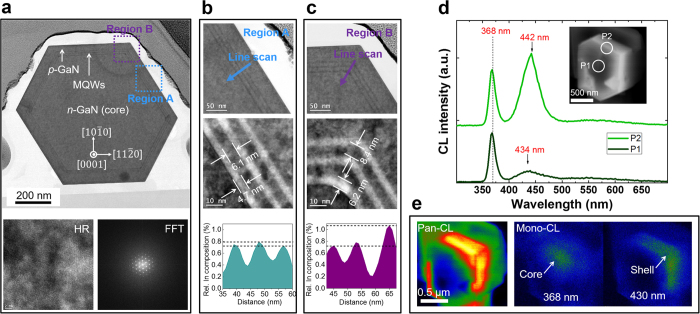
(**a**) Plan-view TEM image of a single core–shell NR and high-resolution image of the core GaN region (left bottom) and its FFT image (right bottom). Enlarged TEM images (upper and middle) and relative indium compositions of region A (**b**) and region B (**c**). (**d**) Spot-mode CL spectra at points P1 and P2 of the SEM image (inset). (**e**) Panchromatic (left) and monochromatic (right) CL emission images at 368 and 430 nm. Arrows in (**b**) and (**c**) indicate the scanned lines in the EDS measurement.

Figure 7a shows a TEM image of the in-plane core–shell NR. The *n*-GaN core region was uniformly wrapped by the shell layers, including the MQWs and the *p*-GaN layer. In particular, no dislocations were propagated into the core regions, as observed in the cross-sectional view. A high-resolution TEM image and its FFT pattern are shown at the bottom left and bottom right panels, respectively, of Fig. 7a. The clear atomic arrangement and six-fold symmetry on the plane view further confirm the high crystal quality of GaN NRs. Although IDBs appeared in the GaN NRs (as evident from Fig. 6a), 2D structural defects such as GBs were absent. TEM enlargements of two representative regions, i.e. region A (all *m*-direction components) and region B (including *a*-direction components), are presented in Fig. 7b,c, respectively. Note that regions A and B resemble quantum well and quantum wire regions, respectively, owing to their different geometries and indium incorporations^48^. Within the same growth time, the thickness of MQWs was more than ~1.3 times higher in region B than in region A, as shown in the middle panels of Fig. 7b,c. This result is consistent with the promoted adatom reaction in the inter-plane regions^49^. The relative indium composition of regions A and B is displayed in the lower panels of Fig. 7a and b, respectively. The actual indium composition in these regions obtained by calibration was ~9.3% even though there is a broad spatial indium variation along the sidewall of core–shell NRs. We additionally observed higher indium compositions and fluctuations in region B than in region A. This result confirms the strong effect of geometric architecture on indium localisation.

The spatial luminescence of the in-plane rod surfaces was also characterised by CL measurements. Figure 7d shows the spot-mode CL spectra measured on the MQWs above core (P1) and shell (P2) regions of the SEM image (inset of Fig. 7d). The CL wavelength and intensity are both slightly higher in P2 than in P1. We have already confirmed that the uppermost surfaces of the core–shell NRs are severely deformed above the IDB regions. This growth phenomenon resulted in parasitic pyramidal structures that weakened the CL emission intensity on the top surface of the core–shell NRs. Figure 7e presents the panchromatic (left) and monochromatic CL (right) images acquired at emission wavelengths of 368 and 430 nm. Relatively prominent emission occurred in the core regions at 368 nm and in the shell regions at 430 nm, indicating optically active occupation of core (GaN) and shell (InGaN/GaN MQWs), respectively. Hence, MQW-related CL emission featured compositionally localised behaviours at around sidewalls with high CL intensity.

### Photoluminescence of InGaN/GaN core–shell NR array

**Figure 8:**
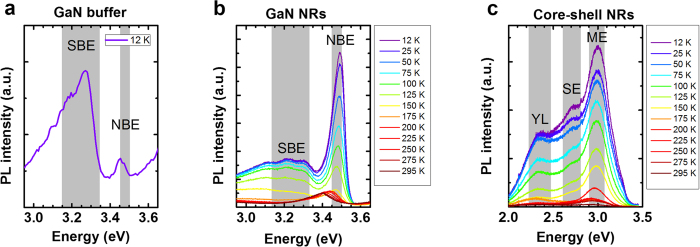
Temperature-dependent PL spectra. (**a**) PL spectrum of the GaN buffer at 12 K. PL spectra of (**b**) GaN NRs and (**c**) InGaN/GaN core–shell NRs at 12–295 K range.

We then investigated the detailed optical properties of the NRs by temperature-dependent photoluminescence (PL) measurements. The results are presented in Fig. 8. Here we analysed the NRs grown at 1060 °C and the subsequent core–shell NRs, which yielded the most intense band edge emissions at room temperature (see Supplementary Fig. S4). The PL was measured on NR ensembles that were properly grown on the designated opening area (see Supplementary Fig. S5). Figure 8a shows the PL spectrum of the GaN buffer at low temperature (12 K). Spectra at higher temperatures are not shown because their intensities were weak. The NBEs and sub-bandgap emissions (SBEs) (grey areas in Fig. 8a) were 3.45–3.50 eV and 3.15–3.3 eV, respectively. The GaN buffer exhibited a weak NBE peak at 3.45 eV and a prominent SBE peak at 3.27 eV. Considering the chemical composition of the quartz substrates and the low crystal quality of the GaN buffer, the NBE peak at 3.45 eV is assigned to the excitons bound to point defect levels induced by Si and O^50^. The SBE peak at 3.27 eV is assigned to donor–acceptor pair (DAP) recombination. As is well known, DAP transitions in GaN nanostructures are enhanced by strong inter-diffusion of Si^51^,^52^. By elongating the GaN NRs to heights of several micrometres, we considerably suppressed but did not eliminate the DAP emission, as evidenced by the broad and weak peaks in the SBE area of Fig. 8b. The low DAP emission of the GaN NRs might be attributed to decomposed Si from the SiO_2_ masking layer and to parasitic DAP emission from the GaN buffer. On the other hand, the dramatically enhanced NBE confirms the improved crystal quality. The NBE of the GaN NRs peaked at 3.49 eV, slightly higher than the typical peak of neutral donor-bound excitons (D^0^X), which appears at 3.47 eV. This slight increase might arise from compressive strain of the GaN NRs as well as Si impurities dissociated from the mask^53^,^54^,^55^. The compressive strain of GaN NRs was confirmed by Raman spectroscopy (see Supplementary Fig. S6). According to the TEM analysis, the broadening of the NBE into lower energies might be partially attributed to IDB-related emission. Recently, the PL emission peak of GaN NRs at low temperatures (3.45 eV) has been attributed to IDB-related defects^56^. Moreover, the *I*_2_-type basal-plane SFs, which emit at 3.35 eV, were clearly suppressed in the GaN NRs. High micro scale SF densities, which appear in GaN layers grown on amorphous substrates, are known to degrade the NBE of GaN^57^. However, as evident in the TEM observations (Fig. 7a), our fabricated GaN NRs were almost SF-free. The heavily suppressed PL transition at 3.35 eV also confirms the structural integrity of our GaN NRs and highlights the excellent advantage of nanoscale local epitaxy over micro scale epitaxy. The temperature dependence of the peak energies of various transitions in GaN NRs, and the intensities of the donor-bound exciton transitions, further support the excellence of nanoscale local epitaxy (see Supplementary Fig. S7). The D^0^X peak energies of GaN NRs fabricated at 1060 °C (Fig. S7(a)) decreased with increasing temperature and were well fitted to Varshni’s equation^58^. Moreover, the DAPs and their phonon replicas were related to their optical depths (222–225 meV)^59^. The PL intensities of the D^0^X lines of the GaN NRs presented two activation energies at different temperatures. Below 50 K, the D^0^X lines were quenched by weak activation energies in the 7–9 meV range. These energies well correspond to the optical binding energy of D^0^X^59^. Above 50 K, the D^0^X lines were quenched by the second activation energies of 34–36 meV, corresponding to the sum of the D^0^X binding energy and the activation energy of free excitons^60^. Therefore, the activation energies of free excitons in GaN NRs ranged from 26 to 28 meV, approximating the binding energy of the Si donor (22.8 meV)^61^.

Figure 8c shows the PL spectra of the InGaN/GaN core–shell NRs. The broad QW emission was attributed to mixed *m*-plane emission (ME), semipolar emission (SE) and yellow luminescence (YL). YL is commonly found on GaN epilayers and originates from structural imperfection^62^,^63^. Here, we selected a growth temperature that optimised the morphology of the grown NRs (Fig. 2) and hence reduced the YL emission (see Supplementary Fig. S4). The polar-plane emission on the top surfaces was not assigned because of weak contribution to the total QW emission, as evident in CL mapping (Figs 6e and 7e). Instead, SE might include the emissive contribution from the newly formed pyramidal structures of the deformed polar plane (see Fig. 7). Of course, the ME was spectrally broadened by the thickness (top-to-bottom) variation of the *m*-plane MQW layers. The ME peaks monotonically blue-shifted with decreasing temperature, consistent with Varshni’s empirical model^58^. The total peak energy of the ME monotonically shifted by 89 meV, also reasonably reflecting the potential gradient behavior^64^,^65^. This potential gradient tends to hinder the typical temperature-dependent S-shaped curves, originated from deep indium localisations^66^,^67^,^68^. Similarly, the SE peak energy shifted by 87 meV, indicating another potential gradient of the semipolar facets. The indium distribution in InGaN/GaN core–shell layers is often spatially variable, because the rate of indium incorporation depends on the surface energy. Therefore, the achieved crystal quality of GaN NRs on amorphous substrates and the radiative emission properties of the core–shell structures are suitably pristine for state-of-the-art nanoscale 3D optoelectronics.

## Conclusion

By combining MBE and MOCVD with nanoscale local epitaxy, we grew high-quality GaN NRs on quartz substrates. First, the MBE process was used to grow a relatively flat GaN buffer. The GaN NRs were then selectively elongated by pulsed-mode MOCVD using a dielectric hole array. Despite lacking a POL, the combined system yielded GaN NRs with near-perfect preferential orientation along the surface normal direction. As a quartz substrate has lower thermal conductivity and higher thickness than conventional sapphire substrates, we could determine the appropriate growth temperature that formed uniform NRs by the pulsed-mode growth procedure. The high quality of the grown GaN NRs was demonstrated in various ways. To our knowledge, we present the first demonstration of micrometre-long GaN NR array on amorphous layers using MOCVD. In the grown GaN NRs, not only were the SF-related defect emissions innovatively suppressed but the NBE emissions were also improved, as evidenced by the clear D^0^X emission. Furthermore, the outer shell layers of InGaN/GaN grown around the GaN core region presented a spatial potential gradient along the growth axis, similarly to normal core–shell 3D NRs grown by homoepitaxy. To improve the crystal quality and preferential orientation, we could insert a POL before growing the buffer layer. With this improvement, our fabricated NRs could be employed in novel device applications on amorphous layers.

## Methods

### Template preparation

To induce pre-orienting behaviour, we first grew a GaN buffer layer by MBE. In this process, a 2-inch quartz substrate mounted on an indium-free block was loaded in the preparation chamber of modified PA-MBE (VG-V80). After pre-outgassing at 300 °C for 30 min, the sample was transferred to the growth chamber and thermally annealed at 900 °C for 30 min. Next, nitridation was performed at 700 °C for 1 h at an RF plasma power of 350 W and an N_2_ flow of 1.0 sccm. The nitridation process assists to form silicon nitride, thereby improving morphology and crystallinity of GaN layer on amorphous templates^11^,^69^. Afterwards, a GaN layer was grown at 800 °C with a Ga beam equivalent pressure of 3 × 10^−5^ mbar. During the GaN growth, the chamber pressure was maintained at 3 × 10^−7^ mbar and the real-time information of the grown layer (thickness, crystal quality and orientation) was monitored by *in-situ* reflection high-energy electron diffraction (Arios 201). The MBE-grown GaN buffer was ~250 nm thick. To perform the nanoscale SAG, a dielectric hole array was formed on the MBE-grown GaN buffer layer. A 30-nm-thick SiO_2_ layer was deposited on the GaN buffer/quartz template by RF magnetron sputtering (ULVAC, ACS-4000-UHV-C3). A circular hole array with a polymer resist (Micro resist technology, MR-I 7020E imprint polymer) was then formed by a thermal nanoimprint system (Scivax, X-500). Subsequently, the circular hole array was extended from the patterned resist to the SiO_2_ layer by reactive ion etching (RIE). Finally, the resist mask was cleaned in acetone. Then, a piece (1 cm × 1 cm) was cut from the fabricated SiO_2_ hole array template using a dicing machine (Accretech, AWD-100A).

### Growth of core–shell NRs

The GaN NR array was grown on the SiO_2_ hole array templates by pulsed-mode MOCVD. The pulsed-mode process cycled through four steps: trimethylgallium (TMG) ON (*t*_*1*_), NH_3_ ON (*t*_*2*_), post-TMG OFF (*t*_*3*_) and post-NH_3_ OFF (*t*_*4*_). For the ON steps, *t*_*1*_ was 5 s with a TMG flow of 15 sccm (78 μmol/min), and *t*_*2*_ was 15 s with an NH_3_ flow of 5 slm (223.21 mmol/min). For the OFF steps, *t*_*3*_ and *t*_*4*_ were fixed at 1 s and pure H_2_ was injected to maintain a constant total gas flow. Tetramethylsilane (Si(CH_3_)_4_) was injected with a molar flow of 2.4 nmol/min, leading to *n*-type carrier concentration of ~1.5 × 10^18^ cm^−3^. The growth temperature was controlled within 1020–1080 °C. All GaN NR structures were grown through 150 cycles. Afterwards, InGaN/GaN MQWs were grown at 770 °C in ambient nitrogen. Here, the Ga, In and N were sourced from TMG, trimethylindium and NH_3_, respectively. The growth times of the InGaN quantum well and GaN barrier were 1.5 and 5 min, respectively. Finally, the outermost layer was capped with Mg-doped *p*-GaN at 900 °C for 6 min, grown under conventional continuous-mode growth in ambient H_2_. At this growth stage, the flow rates of TMG and NH_3_ were adjusted to 20 sccm and 8 slm, respectively. As a precursor, bis-ethylcyclopentadienyl magnesium (EtCp_2_Mg) was used with a molar flow of 240 nmol/min, which typically leads to *p*-type carrier concentration of ~2 × 10^17^ cm^−3^ for a planar *p*-GaN layer with Hall measurement. Throughout the growth process, the reactor pressure was maintained at 200 Torr.

### Structural and optical characterisations

The surface morphology of the samples was observed by field emission SEM (FE-SEM; Hitachi, SU70). The crystalline orientations were characterised by EBSD (Oxford Instruments, INCA Crystal EBSD system) in the FE-SEM at 20 kV with a working distance of 27 mm and a sample tilt of 70°. The specimen was fabricated by a dual-beam focused ion beam (NOVA 200), and its microstructural properties were examined by TEM (JEOL, ARM200F). A compositional analysis was performed by EDS equipped with the TEM. The temperature-dependent PL was measured with a 325-nm He–Cd laser. The PL set-up was equipped with a laser spot diameter of 80 μm and a power density of 0.2 kW/cm^−2^. It was composed of a normal lens with a numerical aperture of 0.125 and a focal length of 100 mm. The spatial emission properties were studied by spot-mode CL (Gatan, MONO CL3+) and monochromatic emission mapping.

## Additional Information

**How to cite this article**: Bae, S.-Y. *et al*. III-nitride core–shell nanorod array on quartz substrates. *Sci. Rep.* **7**, 45345; doi: 10.1038/srep45345 (2017).

**Publisher's note:** Springer Nature remains neutral with regard to jurisdictional claims in published maps and institutional affiliations.

## Supplementary Material

Supplementary Information
