# Supplementary material for: III-nitride core–shell nanorod array on quartz substrates
Source: Sci Rep. 2017 Mar 27;7:45345. doi: 10.1038/srep45345 (PMC5366955; doi:10.1038/srep45345)
Supplement: Supplementary Information [file srep45345-s1.pdf]

Supplementary Information

III-nitride core–shell nanorod array on quartz substrates

Si-Young Bae[1,*,+], Jung-Wook Min[2,+], Hyeong-Yong Hwang[2], Kaddour Lekhal[1,], Ho-Jun Lee[3], Young-Dahl Jho[2], Dong-Seon Lee[2], Yong-Tak Lee[2], Nobuyuki Ikarashi[1], Yoshio Honda[1] & Hiroshi Amano[1,4]**

[1] Institute of Materials and Systems for Sustainability (IMaSS), Nagoya University, Nagoya, 464-8603, Japan
[2] School of Electrical Engineering and Computer Science, Gwangju Institute of Science and Technology (GIST), Gwangju, 61005, Republic of Korea
[3] Department of Electrical Engineering and Computer Science, Nagoya University, Nagoya, 464-8603, Japan
[4] Akasaki Research Center (ARC), Nagoya University, Nagoya, 464-8603, Japan
[*] siyoubae@gmail.com
[**] lekhal.kaddour@yahoo.fr
[+] these authors contributed equally to this work

1. Amorphous substrates and pre-orienting layer (POL)

<table>
<tr><td>Amorphous substrates</td><td colspan="6">· Insulator: Glass and quartz (fused silica)[1]
· Metal: Mo, Ta, Nb, Al and Ag[2–4]</td></tr>
<tr><td>POL</td><td colspan="6">Preferential orientation along out-of-plane and random in-plane orientations
· Cu, Ti, Pt, Ni, Hf, Zr and Graphene[5–10]</td></tr>
<tr><td rowspan="4">Crystal structure
Lattice (Å)
(Mismatch with GaN)</td><td>Mo</td><td>Ta</td><td>Nb</td><td>Al</td><td>Ag</td><td>Cu</td></tr>
<tr><td>BCC
a = 3.147
(−1.34%)</td><td>BCC
a = 3.301
(3.47%)</td><td>BCC
a = 3.300
(3.44%)</td><td>FCC
a = 4.049
(26.92%)</td><td>FCC
a = 4.085
(28.05%)</td><td>FCC
a = 3.614
(13.29%)</td></tr>
<tr><td>Ti</td><td>Pt</td><td>Ni</td><td>Hf</td><td>Zr</td><td>Graphene</td></tr>
<tr><td>HCP
a = b =
2.950
(−7.52%)</td><td>FCC
a = 3.924
(23.00%)</td><td>FCC
a = 3.524
(10.47%)</td><td>HCP
a = b =
3.196
(0.18%)</td><td>HCP
a = b =
3.232
(1.31%)</td><td>Hexagonal
a = b =
3.230
(1.29%)</td></tr>
</table>

Table S1. Candidates of amorphous substrates and pre-orienting layers. Crystal structures, in-plane lattice and in-plane lattice mismatches with GaN are listed.

2. Factors affecting GaN growth and fabrication on amorphous substrates

Factors	Values (or requirements)	Proposed solution
Glass-transition temperature (°C)	Soda-lime glass (< 600) and quartz (< 1200)	Sputtering (< 600)[9]
Thermal expansion coefficient ($\times 10^{-6}$ / K) [α_{GaN} = 6.5]	▪ Substrate: Quartz (0.33), Mo (4.8), Ta (6.3), Nb (7.3), Al (23.1), Ag (18.9)<br>▪ POL: Cu (16.5), Ti (8.6), Pt (8.8), Ni (13.4), Hf (5.9), Zr (5.7), Graphene (−8)	Strain compensation buffer or selective area growth (SAG)[11,12]
Thermal conductivity ($W \cdot m^{-1} \cdot K^{-1}$)	▪ Substrate: Quartz (1.4), Mo (139), Ta (57), Nb (54), Al (235), Ag (430)<br>▪ POL: Cu (400), Ti (22), Pt (72), Ni (91), Hf (23), Zr (23), Graphene (> 500)	Transfer to metal substance[13]
Crystal quality	XRD_{FWHM} < ~400 arcsec (on amorphous substrates)	Evolutionary selection SAG (ES-SAG) or nanowires[14,15]
Optical property	PL (D^0X) = ~3.47 eV and Raman [E_2(h)] = 567.0 ± 0.1 cm^{-1}	POL or nanowires[15,16]
Electrical operation	Formation of p–n current-injection electrodes	Contact after substrate removal or metal-based POL[16,17]

Table S2. Important factors, values (requirements) and proposed solutions for device operation on amorphous substrates.

3. Estimation of the pitch-to-pitch distance (L) on the mask hole array

After calculating the tilt angle (θ) and radius (r) of the GaN NRs from their statistical distribution, we estimated the acceptable minimum of the pitch-to-pitch distance of the mask holes (L). Although the height (h) of the NRs depends on the growth condition, it can be adjusted by controlling the growth time. Assuming two adjacent NRs with equivalent height (see Supplementary Fig. S1), the acceptable distance between the hole masks is simply determined as

$$L = 2(l_1 + l_2) = 2(h\sin\theta + r\cos\theta),$$

where l_1 and l_2 are distances determined by h and r, respectively.

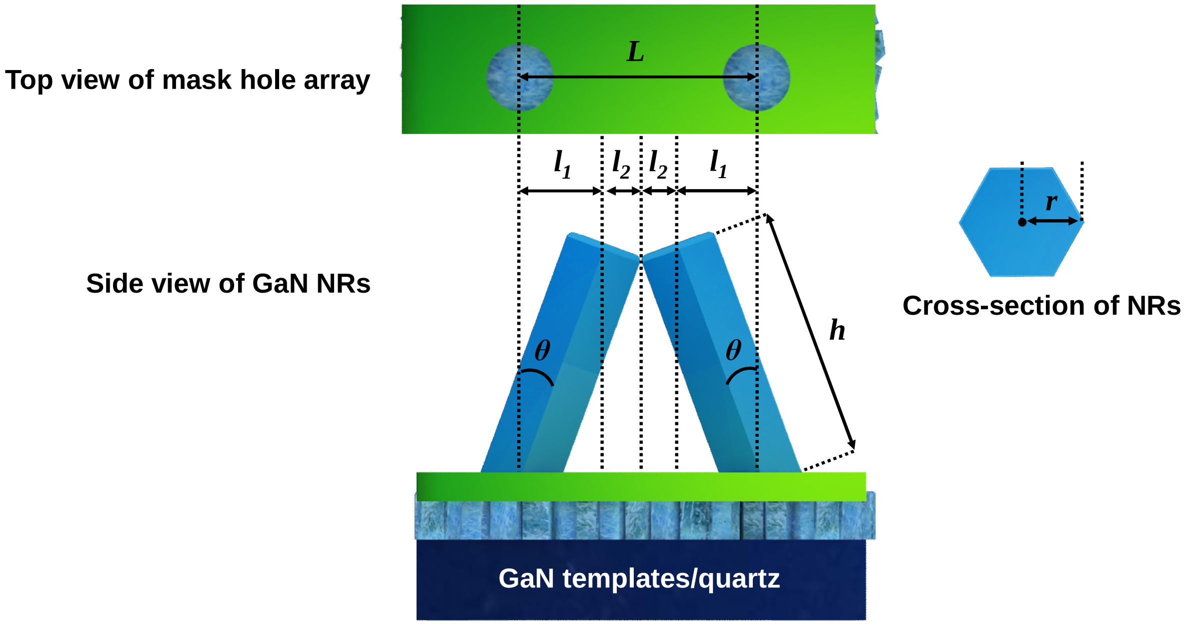

Figure S1. Schematic of GaN NRs for determining the acceptable minimum of L.

4. Specimen preparation of InGaN/GaN core–shell nanorods for TEM measurement

The as-grown NRs were broken by sonication in isoprophylene alcohol for 10 s, and the separated NRs were dropped onto planar substrates. After selecting appropriate samples, the samples were passivated by Pt or W coating. The samples were then sliced by a focused ion beam (FIB), forming in-plane and out-of-plane cuts as shown in Fig. S2.

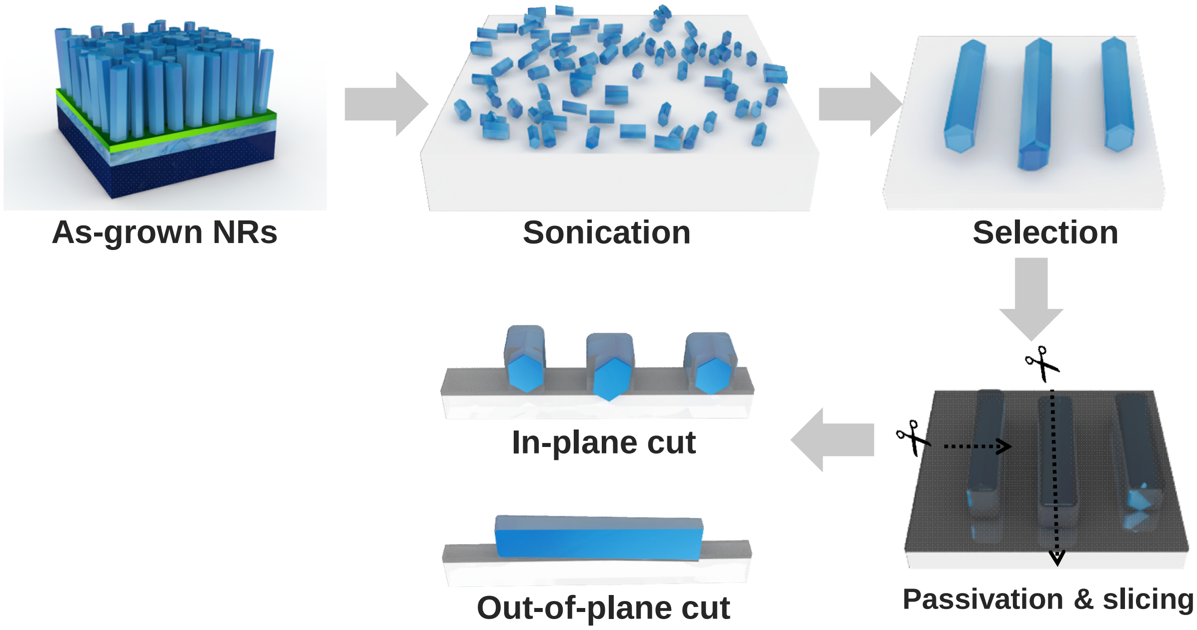

Figure S2. Schematic of the FIB procedure for preparing TEM specimens. In-plane and out-of-plane cuts of InGaN/GaN core–shell NRs were obtained.

5. KOH etching test of GaN nanorods

To study the polarity of the grown GaN NRs, we applied chemical etching with 4 M KOH solution at 44 °C. As-grown GaN NRs formed at 1040 °C are shown in Fig. S3(a). As the etching time increased, obvious pyramidal structures appeared on the top surfaces, indicating large N polarity in the grown GaN NRs. In fact, the partially etched NRs are dominated by mixed polarity.

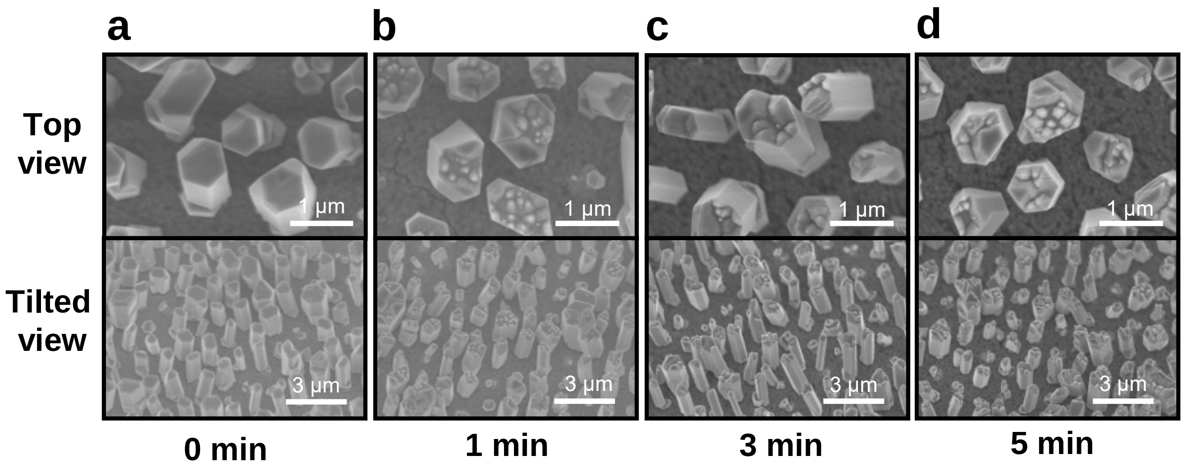

Figure S3. SEM images of GaN NRs with KOH etching times of (a) 0 min (before etching), (b) 1 min, (c) 3 min and (d) 5 min. Upper and lower images present top and tilted views, respectively.

6. PL spectra of GaN NRs at room temperature

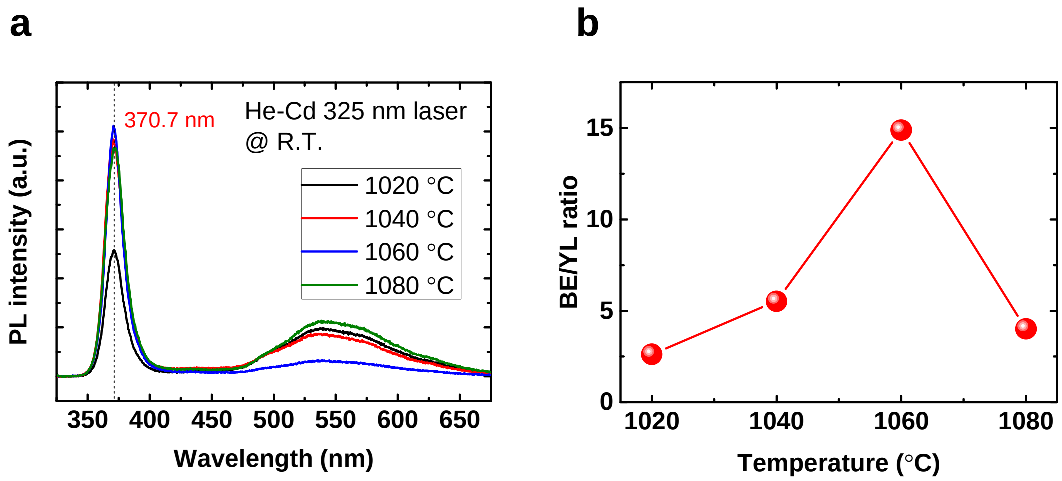

Figure S4. (a) PL spectra of GaN NRs grown at various growth temperatures and (b) their BE/YL ratios. The BE/YL ratio was maximised at 1060 °C. Therefore, all further studies of GaN NRs with core–shell layers were based on GaN NRs grown at 1060 °C.

7. Surface morphology of InGaN/GaN core–shell nanorods

Figure S5 shows the change in morphology from GaN NRs to InGaN/GaN core–shell NRs. GaN NRs were selectively grown over a large area (hundreds of micrometres), as shown in Fig. S5(a). At a higher magnification (Fig. S5(b), dense, elongated GaN NRs are seen on the flat top surfaces. The top surfaces of the NRs were severely structurally deformed during the shell growth (Fig. S5(c)).

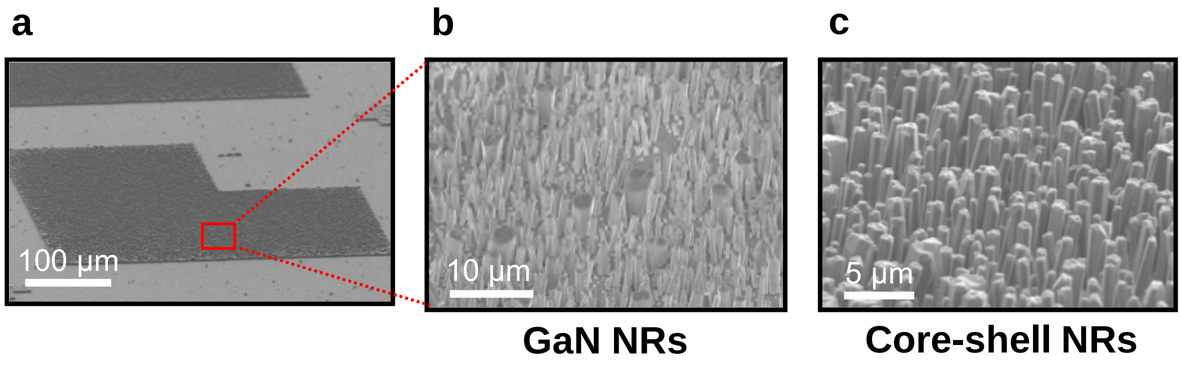

Figure S5. SEM images of (a, b) GaN NRs and (c) InGaN/GaN core–shell NRs.

8. Raman spectra of GaN NRs

Figure S6 shows the Raman spectra of GaN NRs grown at 1020–1080 °C. The E_2(h) of all samples peaked around 568.5 cm^{-1}. As the E_2(h) of strain-free GaN is ~567.0 ± 0.1 cm^{-1}, this result indicates compressive strain[18]. Although A_1(LO) was only observed in GaN NRs grown at 1040 °C, its frequency (738.5 cm^{-1}) was higher than that in strain-free GaN (~736.5 cm^{-1}), consistent with the compressive strain behaviours inferred from the E_2(h) peaks.

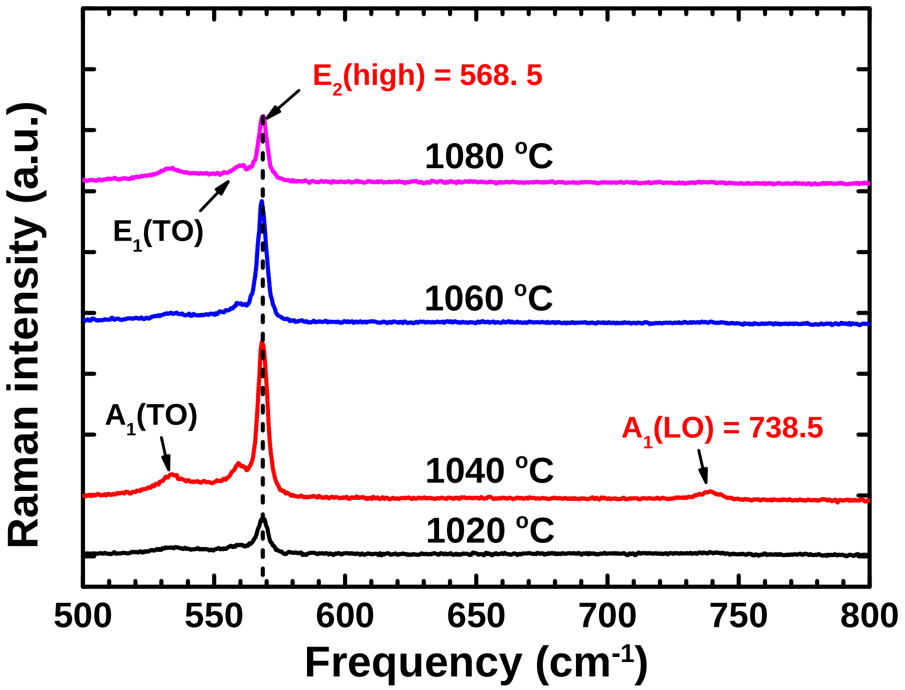

Figure S6. Raman spectra of GaN NRs grown at 1020–1080 °C. All samples featured compressive strains.

9. Temperature dependences of peak energies of various transitions and PL intensities of D^0X

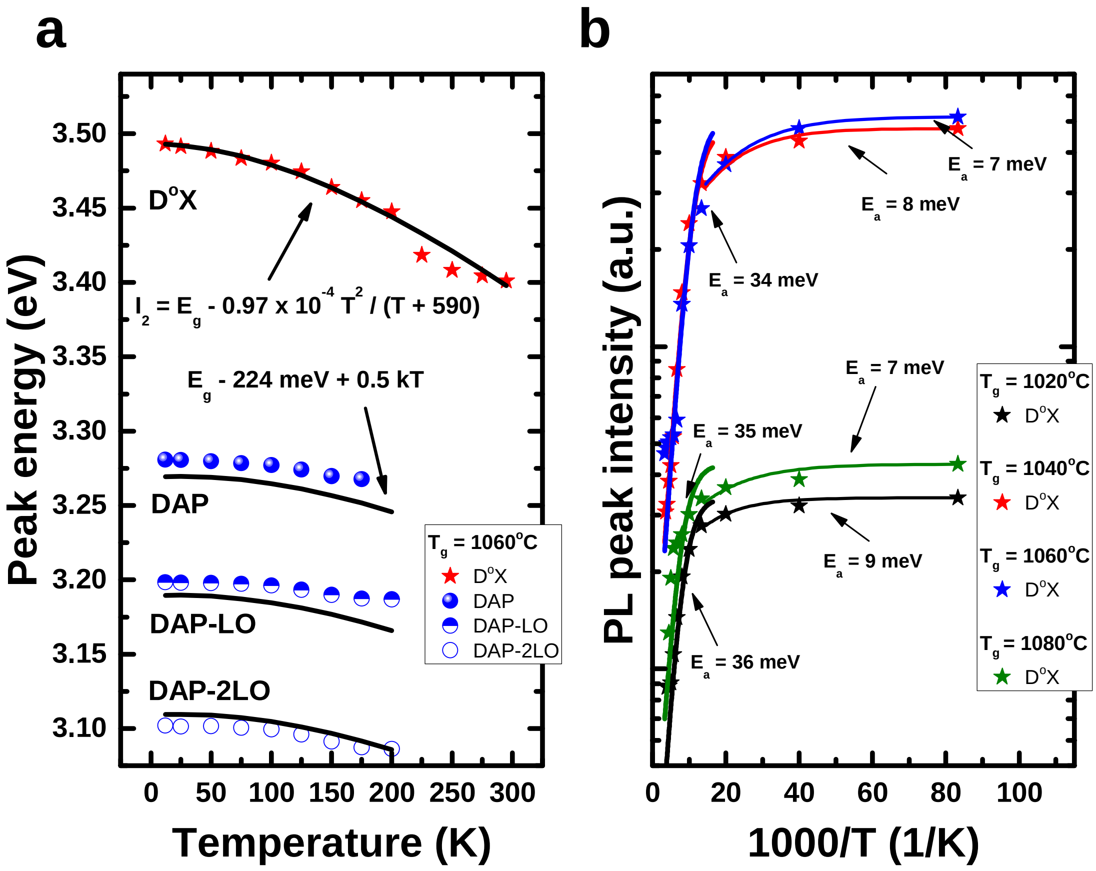

Figure S7. (a) Temperature dependence of the peak energies of various transitions in GaN NRs grown at 1060 °C and (b) intensities of the donor bound exciton transitions in GaN NRs grown at 1020–1080 °C.

References

1. Hiroki, M., Asahi, H., Tampo, H., Asami, K. & Gonda, S. Improved properties of polycrystalline GaN grown on silica glass substrate. *J. Cryst. Growth* **209,** 387–391 (2000).
2. Yamada, K. *et al.* Strong photoluminescence emission from polycrystalline GaN layers grown on W, Mo, Ta, and Nb metal substrates. *Appl. Phys. Lett.* **78,** 2849–2851 (2001).
3. Inoue, S., Okamoto, K., Nakano, T., Ohta, J. & Fujioka, H. Growth of single crystalline GaN on silver mirrors. *Appl. Phys. Lett.* **91,** 201920 (2007).
4. Zhao, C. *et al.* Facile formation of high-quality InGaN/GaN quantum-disks-in-nanowires on bulk-metal substrates for high-power light-emitters. *Nano Lett.* **16,** 1056–1063 (2016).
5. Qin, F.-W. *et al.* Growth of high c-orientated crystalline GaN films on amorphous Cu/glass substrates with low-temperature ECR-PEMOCVD. *J. Mater. Sci. Mater. Electron.* **25,** 969–973 (2014).
6. Wolz, M. *et al.* Epitaxial growth of GaN nanowires with high structural perfection on a metallic TiN film. *Nano Lett.* **15,** 3743–3747 (2015).
7. Zhong, M. M. *et al.* Low-temperature growth of high c-orientated crystalline GaN films on amorphous Ni/glass substrates with ECR-PEMOCVD. *J. Alloys Compd.* **583,** 39–42 (2014).
8. Sarwar, A. T. M. *et al.* Semiconductor nanowire light-emitting diodes grown on metal: a direction toward large-scale fabrication of nanowire devices. *Small* **11,** 5402–5408 (2015).
9. Shon, J. W., Ohta, J., Ueno, K., Kobayashi, A. & Fujioka, H. Fabrication of full-color InGaN-based light-emitting diodes on amorphous substrates by pulsed sputtering. *Sci. Rep.* **4,** 5325 (2014).
10. Chae, S. J. *et al.* Direct growth of etch pit-free GaN crystals on few-layer graphene. *RSC Adv.* **5,** 1343–1349 (2015).
11. Cosendey, G., Carlin, J.-F., Kaufmann, N. A., Butté, R. & Grandjean, N. Strain compensation in AlInN/GaN multilayers on GaN substrates: Application to the realization of defect-free Bragg reflectors. *Appl. Phys. Lett.* **98,** 181111 (2011).
12. Zheleva, T. S., Nam, O.-H., Ashmawi, W. M., Griffin, J. D. & Davis, R. F. Lateral epitaxy and dislocation density reduction in selectively grown GaN structures. *J. Cryst. Growth* **222,** 706–718 (2001).
13. Chung, K., Lee, C.-H. & Yi, G.-C. Transferable GaN layers grown on ZnO-coated graphene layers for optoelectronic devices. *Science* **330,** 655–657 (2010).
14. Leung, B., Song, J., Zhang, Y. & Han, J. Evolutionary selection growth: towards template-insensitive preparation of single-crystal layers. *Adv. Mater.* **25,** 1285–1289 (2013).
15. Zhao, S., Kibria, M. G., Wang, Q., Nguyen, H. P. T. & Mi, Z. Growth of large-scale vertically aligned GaN nanowires and their heterostructures with high uniformity on SiO x by catalyst-free molecular beam epitaxy. *Nanoscale* **5,** 5283–5287 (2013).
16. Choi, J. H. *et al.* Nearly single-crystalline GaN light-emitting diodes on amorphous glass substrates. *Nat. Photonics* **5,** 763–769 (2011).
17. Choi, J. H. *et al.* Fully flexible GaN light-emitting diodes through nanovoid-mediated transfer. *Adv. Opt. Mater.* **2,** 267–274 (2014).
18. Goni, A. R., Siegle, H., Syassen, K., Thomsen, C. & Wagner, J.-M. Effect of pressure on optical phonon modes and transverse effective charges in GaN and AlN. *Phys. Rev. B* **64,** 035205 (2001).
